# Study of weak solutions for parabolic variational inequalities with nonstandard growth conditions

**DOI:** 10.1186/s13660-018-1623-0

**Published:** 2018-02-08

**Authors:** Yan Dong

**Affiliations:** Department of Basic, Shaanxi Railway Institute, Weinan, China

**Keywords:** 35B40, 35K35, Parabolic variational inequality, Weak solution, Penalty method, Existence

## Abstract

In this paper, we study the degenerate parabolic variational inequality problem in a bounded domain. First, the weak solutions of the variational inequality are defined. Second, the existence and uniqueness of the solutions in the weak sense are proved by using the penalty method and the reduction method.

## Introduction

This article concerned with initial-boundary problem whose model is

1 $$ \textstyle\begin{cases} \min\{ Lu,u(x,0) - {u_{0}}(x)\} = 0,& (x,t) \in{Q_{T}},\\ u(x,t) = 0,& (x,t) \in{\Gamma_{T}},\\ u(x,0) = {u_{0}}(x),& x \in\Omega, \end{cases} $$

with

$$Lu = {u_{t}} - \operatorname{div}\bigl( {a(u){{ \vert {\nabla u} \vert }^{p(x,t) - 2}}\nabla u} \bigr) - f(x,t),\quad a(u) = {u^{\sigma}} + {d_{o}}, $$

where $\Omega \subset{\mathbb{R}^{+} }$ is a bounded simply connected domain, ${Q_{T}} = \Omega \times(0,T]$, and ${\Gamma_{T}}$ denotes the lateral boundary of the cylinder ${Q_{T}}$.

This type of variational inequality was studied initially by Chen and Yi [1], who proposed the equation

2 $$ \textstyle\begin{cases} \frac{\partial}{{\partial\tau}}V - \frac{1}{2}{\sigma^{2}}\frac {{{\partial^{2}}}}{{\partial{x^{2}}}}V - ( {r - \frac{1}{2}{\sigma^{2}}} )\frac{\partial}{{\partial x}}V + rV \ge0 &\text{in } {\Omega_{T}},\\ V \ge g(x), &\text{in } {\Omega_{T}},\\ ( {\frac{\partial}{{\partial\tau}}V - \frac{1}{2}{\sigma^{2}}\frac {{{\partial^{2}}}}{{\partial{x^{2}}}}V - ( {r - \frac{1}{2}{\sigma^{2}}} )\frac{\partial}{{\partial x}}V + rV} )(V - g(x)) = 0 &\text{in }{\Omega_{T}},\\ V(t,x) = 0 &\text{on } \partial{\Omega_{T}},\\ V(x,0) = g(x) &\text{in } \Omega \end{cases} $$

for modeling the American option. When *r* and *σ* are positive constant, the existence and uniqueness of solutions to problem (4) were also studied in [2–4].

In 2014, the authors in [5] discussed the problem

$$\textstyle\begin{cases} {u_{t}} - Lu - F(x,t,u,\nabla u) \ge0&\text{in }{Q_{T}},\\ u(x,t) \ge{u_{0}}(x)& \text{in } {Q_{T}},\\ ( {{u_{t}} - Lu - F(x,t,u,\nabla u)} )(u - {u_{0}}(x)) = 0 &\text{in } {Q_{T}},\\ u(x,0) = {u_{0}}(x) &\text{on } \Omega,\\ u(x,t) = g(x) &\text{on } \partial\Omega \times(0,T) \end{cases} $$

with second-order elliptic operator

$$L(x,t) = \sum_{i,j = 1}^{d} { \frac{\partial}{{\partial{x_{j}}}}} \biggl( {{a^{ij}}(x,t)\frac{\partial}{{\partial{x_{i}}}}} \biggr) - \sum_{i,j = 1}^{d} {{b^{i}}(x,t) \frac{\partial}{{\partial{x_{i}}}}} - c(x,t). $$

They proved the existence and uniqueness of a solution to this problem with some conditions on ${u_{0}}$, *F*, and *L*. Later, the authors in [6, 7] extended the relative conclusions with the assumption that $a(u)$ and $p(x)$ are two positive constants. The author discussed the existence and uniqueness of a solution by the penalty method.

The existence and uniqueness of such a problem with the assumption that $p(x)$ and $a(u)$ are variables were less studied.

The aim of this paper is to study the existence and uniqueness of solutions for a degenerate parabolic variational inequality problem. Throughout the paper, we assume that the exponent $p(x,t)$ is continuous in $Q = \overline{{Q_{T}}} $ with logarithmic module of continuity:

3 $$\begin{aligned}& 1 < {p^{-} } = \inf_{(x,t) \in Q} p(x,t) \le p(x,t) \le{p^{+} } = \sup _{(x,t) \in Q} p(x,t) < \infty, \end{aligned}$$

4 $$\begin{aligned}& \forall z = (x,t) \in Q,\qquad \xi = (y,s) \in{Q_{T}},\qquad \vert {z - \xi} \vert < 1,\qquad \bigl\vert {p(z) - p(\xi)} \bigr\vert \le\omega \bigl( \vert {z - \xi} \vert \bigr), \end{aligned}$$

where

$$\mathop{\lim\sup}\limits _{\tau \to{0^{+} }} \omega(\tau)\ln\frac {1}{\tau} = C < + \infty. $$

The outline of this paper is as follows. In Section 2, we introduce the function spaces of Orlicz-Sobolev type, give the definition of a weak solution to the problem, and prove the existence and uniqueness. Section 3 is devoted to the proof of the existence and uniqueness of the solution obtained in Section 2.

## Basic spaces and the main results

To study our problems, let us introduce the Banach spaces:

$$\begin{aligned}& {L^{p(x,t)}}({Q_{T}}) = \biggl\{ u(x,t) \Big\vert u \text{ is measurable in } {Q_{T}},{A_{p(\cdot)}}(u) = \int {\int_{{Q_{T}}} {{{\left| u \right|}^{p(x,t)}}\, {\mathrm{d}}x\, {\mathrm{d}}t < \infty } } \biggr\} , \\& { \Vert u \Vert _{p(\cdot)}} = \inf\bigl\{ \lambda > 0,{A_{p(\cdot)}}(u/\lambda) \le1\bigr\} , \\& {V_{t}}(\Omega) = \bigl\{ u \vert {u \in{L^{2}}(\Omega) \cap W_{0}^{1,1}(\Omega)}, \vert {\nabla u} \vert \in {L^{p(x,t)}}(\Omega)\bigr\} ,\qquad { \Vert u \Vert _{{V_{t}}(\Omega)}} = { \Vert u \Vert _{2,\Omega}} + { \vert {\nabla u} \vert _{p(\cdot )\Omega}}, \\& W({Q_{t}}) = \bigl\{ u:[0,T] \mapsto{V_{t}}(\Omega) \vert {u \in {L^{2}}({Q_{t}})}, \vert {\nabla u} \vert \in {L^{p(x,t)}}({Q_{T}}),u = 0\text{ on } {\Gamma_{T}} \bigr\} , \\& { \Vert u \Vert _{W({Q_{t}})}} = { \Vert u \Vert _{2,{Q_{T}}}} + { \vert {\nabla u} \vert _{p(x,t),{Q_{T}}}} \end{aligned}$$

and denote by $W'({Q_{T}})$ the dual of $W({Q_{T}})$ with respect to the inner product in ${L^{2}}({Q_{T}})$.

In spirit of [3] and [4], we introduce the following maximal monotone graph:

$$G(\lambda) = \textstyle\begin{cases} 0,& \lambda > 0,\\ [0,+\infty),& \lambda = 0. \end{cases} $$

In addition, we define the following function class for the solution:

$$B = \bigl\{ {u \in W({Q_{T}}) \cap{L^{\infty}} \bigl(0,T;{L^{\infty}}(\Omega)\bigr)} \bigr\} . $$

### Definition 2.1

A pair $(u,\xi) \in B \times{L^{\infty}}({\Omega_{T}})$ is called a weak solution of problem (1) if (a) $u(x,t) \le{u_{0}}(x)$, (b) $u(x,0) = {u_{0}}(x)$, (c) $\xi \in G(u - {u_{0}})$, (d) for all ${t_{1}},{t_{2}} \in[0,T]$, the following identity holds:

$$\int_{{t_{1}}}^{{t_{2}}} \int_{\Omega}\bigl[u{\varphi_{t}} - \bigl({u^{\sigma}} + {d_{0}}\bigr){{ \vert {\nabla u} \vert }^{p(x,t) - 2}}\nabla u\nabla\varphi + f(x,t)\varphi - \xi\varphi\bigr] \,\mathrm{d}x\,\mathrm{d}t = \int_{\Omega}{u\varphi\,\mathrm{d}x \bigg\vert _{{t_{1}}}^{{t_{2}}}}. $$

The main theorem in this section is the following:

### Theorem 2.1

*Let* $p(x,t)$ *satisfy conditions* (3)*–*(4). *Suppose also that the following conditions hold*:

($H_{1}$)
$\max\{ {1,\frac{{2N}}{{N + 2}}} \} < {p^{-} } < N$, $2 \le\sigma < \frac {{2{p^{+} }}}{{{p^{+} } - 1}}$,
($H_{2}$)
${u_{0}} \ge0$, $f \ge0$, ${ \Vert {{u_{0}}} \Vert _{\infty,\Omega}} + \int_{0}^{T} {{{ \Vert {f(x,t)} \Vert}_{\infty,\Omega}}} \,\mathrm{d}t = K(T) < \infty$.

*Then problem* (1) *has at least one weak solution in the sense of Definition *2.1.

### Theorem 2.2

*Suppose that the conditions in Theorem *2.1 *are fulfilled and* ${p^{+} } \ge2$. *Then problem* (1) *admits a unique solution in the sense of Definition *2.1.

## Proof of the main results

In this section, we consider the family of auxiliary parabolic problems

5 $$ \textstyle\begin{cases} {L_{\varepsilon}}{u_{\varepsilon}} + {\beta_{\varepsilon}}({u_{\varepsilon}} - {u_{0}}) = 0, & (x,t) \in{Q_{T}},\\ u(x,t) = \varepsilon, & (x,t) \in{\Gamma_{T}},\\ u(x,0) = {u_{0}}(x) + \varepsilon, & x \in\Omega. \end{cases} $$

Here, *M* is a positive parameter to be chosen later. Moreover,

$$\begin{aligned}& {L_{\varepsilon}} {u_{\varepsilon}} = {\partial_{t}} {u_{\varepsilon}} - \operatorname{div}\bigl( {{a_{\varepsilon,M}}({u_{\varepsilon}}){{ \vert {\nabla{u_{\varepsilon}}} \vert }^{p(x,t) - 2}}\nabla {u_{\varepsilon}}} \bigr) - f(x,t), \\& 0 < {d_{0}} \le{a_{\varepsilon,M}}(u) = {\bigl(\min\bigl({ \vert u \vert ^{2}},{M^{2}}\bigr) + {\varepsilon^{2}} \bigr)^{\frac{\sigma}{2}}} + {d_{0}} \le \bigl({M^{2}} + 1\bigr) + {d_{0}},\quad 0 < \varepsilon < 1, \end{aligned}$$

and ${\beta_{\varepsilon}}( \cdot)$ is the penalty function satisfying

6 $$ \begin{gathered} 0 < \varepsilon \le1,\qquad {\beta_{\varepsilon}}(x) \in{C^{2}}(R),\qquad {\beta _{\varepsilon}}(x) \le0,\qquad {\beta_{\varepsilon}}(0) = - 1, \\ {\beta'_{\varepsilon}}(x) \ge0,\qquad {\beta''_{\varepsilon}}(x) \le0,\qquad \lim_{\varepsilon \to0} {\beta_{\varepsilon}}(x) = \textstyle\begin{cases} 0, & x > 0,\\ - \infty, & x < 0. \end{cases}\displaystyle \end{gathered} $$

Following a similar method as in [6], we can prove that the regularized problem has a unique weak solution ${u_{\varepsilon}}(x,t) \in W({Q_{T}}) \cap{L^{2}}({Q_{T}})$, ${\partial _{t}}{u_{\varepsilon}}(x,t) \in W'({Q_{T}})$ satisfying the following integral identities:

7 $$ \begin{aligned}[b] & \int_{{t_{1}}}^{{t_{2}}} { \int_{\Omega}{\bigl[{u_{\varepsilon}} {\varphi_{t}} - {a_{\varepsilon}},M({u_{\varepsilon}}){{ \vert {\nabla {u_{\varepsilon}}} \vert }^{p(x,t) - 2}}\nabla{u_{\varepsilon}}\nabla\varphi + f(x,t)\varphi \bigr]\,\mathrm{d}x\,\mathrm{d}t} } \\ &\quad = \int_{{t_{1}}}^{{t_{2}}} { \int_{\Omega}{{\beta_{\varepsilon}}({u_{\varepsilon}} - {u_{0}})\varphi\,\mathrm{d}x\,\mathrm{d}t} } + \int _{\Omega}{{u_{\varepsilon}}\varphi\,\mathrm{d}x} \bigg\vert _{{t_{1}}}^{{t_{2}}} \end{aligned} $$

and

8 $$ \int_{{t_{1}}}^{{t_{2}}} \int_{\Omega}\bigl[({\partial_{t}} {u_{\varepsilon}})\varphi + {a_{\varepsilon,M}}({u_{\varepsilon}}){{ \vert {\nabla u} \vert }^{p(x,t) - 2}}\nabla{u_{\varepsilon}}\nabla \varphi - f(x,t)\varphi + { \beta_{\varepsilon}}({u_{\varepsilon}} - {u_{0}})\varphi\bigr] \,\mathrm{d}x\,\mathrm{d}t = 0. $$

We start with two preliminary results that will be used several times.

### Lemma 3.1

*Let* $M(s) = {| s |^{p(x,t) - 2}}s$. *Then for all* $\xi,\eta \in\,\mathrm{R}^{N}$,

$$\begin{aligned}[b] &\bigl(M(\xi) - M(\eta)\bigr) (\xi - \eta) \\ &\quad \ge \textstyle\begin{cases} {2^{ - p(x,t)}}{ \vert {\xi - \eta} \vert ^{p(x,t)}},& 2 \le p(x,t) < \infty,\\ (p(x,t) - 1){ \vert {\xi - \eta} \vert ^{2}}{({ \vert \xi \vert ^{p(x,t)}} + { \vert \eta \vert ^{p(x,t)}})^{\frac{{p(x,t) - 2}}{{p(x,t)}}}}, & 1 < p(x,t) < 2. \end{cases}\displaystyle \end{aligned} $$

### Lemma 3.2

(Comparison principle)

*Assume that* $2 < \sigma < \frac{{2{p^{+} }}}{{{p^{+} } - 1}}$, ${p^{+} } \ge2$, *and* *u* *and* *v* *are in* $W({Q_{T}}) \cap{L^{\infty}}(0,T;{L^{\infty}}(\Omega))$. *If* ${L_{\varepsilon}}u \ge{L_{\varepsilon}}v$ *in* ${Q_{T}}$ *and if* $u(x,t) \le v(x,t)$ *on* $\partial{Q_{T}}$, *then* $u(x,t) \le v(x,t)$ *in* ${Q_{T}}$.

### Proof

We argue by contradiction. Suppose $u(x,t)$ and $v(x,t)$ satisfy ${L_{\varepsilon}}u \ge{L_{\varepsilon}}v$ in ${Q_{T}}$ and there is $\delta > 0$ such that for $0 < \tau \le T$, $w = u - v > \delta$ on the set ${\Omega_{\delta}} = \Omega \cap\{ x:w(x,t) > \delta\} $, and $\mu({\Omega_{\delta}}) > 0$. Let

$${F_{\varepsilon}}(\xi) = \textstyle\begin{cases} \frac{1}{{\alpha - 1}}{\varepsilon^{1 - \alpha}} - \frac{1}{{\alpha - 1}}{\xi^{1 - \alpha}}& \text{if }\xi > \varepsilon,\\ 0& \text{if }\xi \le\varepsilon, \end{cases} $$

where $\delta > 2\varepsilon > 0$ and $\alpha = \frac{\sigma}{2}$. Let a test-function $\xi = {F_{\varepsilon}}(w) \in Z$ in (8). Then

9 $$ \begin{aligned}[b] 0 &\ge \int{ \int_{{Q_{T}}} {\bigl[{w_{t}} {F_{\varepsilon}}(w) + \bigl({v^{\sigma}} + {d_{0}}\bigr) \bigl({{ \vert {\nabla u} \vert }^{p(x,t) - 2}}\nabla u - {{ \vert {\nabla v} \vert }^{p(x,t) - 2}}\nabla v\bigr)\nabla {F_{\varepsilon}}(w)\bigr]\,\mathrm{d}x \,\mathrm{d}t} } \\ &\quad{}+ \int{ \int_{{Q_{T}}} {\bigl({u^{\sigma}} - {v^{\sigma}} \bigr){{ \vert {\nabla u} \vert }^{p(x,t) - 2}}\nabla u\nabla {F_{\varepsilon}}(w)\,\mathrm{d}x\,\mathrm{d}t} } = {J_{1}} + {J_{2}} + {J_{3}}, \end{aligned} $$

where ${Q_{\varepsilon,\tau}} = {Q_{\tau}} \cap\{ (x,t) \in{Q_{\tau}}| {w > \varepsilon} \} $,

$$\begin{aligned}& {J_{1}} = \int{ \int_{{Q_{T}}} {{w_{t}} {F_{\varepsilon}}(w)\,\mathrm{d}x \,\mathrm {d}t} }, \qquad{J_{2}} = \int{ \int_{{Q_{T}}} {\bigl({u^{\sigma}} - {v^{\sigma}} \bigr){w^{ - \alpha}} {{ \vert {\nabla u} \vert }^{p(x,t) - 2}}\nabla u \nabla w\,\mathrm{d}x\,\mathrm{d}t} }, \\& {J_{3}} = \int{ \int_{{Q_{T}}} {\bigl({v^{\sigma}} + {d_{0}} \bigr){w^{ - \alpha}}\bigl({{ \vert {\nabla u} \vert }^{p(x,t) - 2}} \nabla u - {{ \vert {\nabla v} \vert }^{p(x,t) - 2}}\nabla v\bigr)\nabla w \,\mathrm {d}x\,\mathrm{d}t} }. \end{aligned}$$

Now, let ${t_{0}} = \inf\{ t \in(0,\tau]:w > \varepsilon\} $. Then we estimate ${J_{1}}$, ${J_{2}}$, and ${J_{3}}$ as follows:

10 $$ \begin{aligned}[b] {J_{1}} &= \int{ \int_{{Q_{\varepsilon,\tau}}} {{w_{t}} {F_{\varepsilon}}(w)\,\mathrm{d}x \,\mathrm{d}t} = \int_{\Omega}{\biggl( \int_{0}^{{t_{0}}} {{w_{t}} {F_{\varepsilon}}(w) \,\mathrm{d}t} + \int_{{t_{0}}}^{\tau}{{w_{t}} {F_{\varepsilon}}(w)\,\mathrm{d}t} \biggr)} } \,\mathrm{d}x \\ &\ge \int_{\Omega}{ \int_{\varepsilon}^{w(x,\tau)} {{F_{\varepsilon}}(s)\,\mathrm{d}s \,\mathrm{d}x} \ge} \int_{{\Omega_{\delta}}} { \int _{\varepsilon}^{w(x,\tau)} {{F_{\varepsilon}}(s)\,\mathrm{d}s \,\mathrm{d}x} } \\ &\ge \int_{{\Omega_{\delta}}} {(w - 2\varepsilon)} {F_{\varepsilon}}(2 \varepsilon)\,\mathrm{d}x \ge(\delta - 2\varepsilon){F_{\varepsilon}}(2 \varepsilon)\mu({\Omega_{\delta}}). \end{aligned} $$

Let us first consider the case ${p^{-} } \ge2$. By the first inequality of Lemma 3.1 we get

11 $$ \begin{aligned}[b] {J_{2}} &= \int{ \int_{{Q_{\varepsilon,\tau}}} {\bigl({v^{\sigma}} + {d_{0}} \bigr){w^{ - \alpha}}\bigl({{ \vert {\nabla u} \vert }^{p(x,t) - 2}} \nabla u - {{ \vert {\nabla v} \vert }^{p(x,t) - 2}}\nabla v\bigr)\nabla w \,\mathrm{d}x\,\mathrm{d}t} } \\ &\ge \int{ \int_{{Q_{\varepsilon,\tau}}} {\bigl({v^{\sigma}} + {d_{0}} \bigr){w^{ - \alpha}} {2^{ - p(x,t)}} {{ \vert {\nabla w} \vert }^{p(x,t)}}\,\mathrm{d}x\,\mathrm{d}t} } \\ &\ge{2^{ - {p^{+} }}} \int{ \int_{{Q_{\varepsilon,\tau}}} {\bigl({v^{\sigma}} + {d_{0}} \bigr){w^{ - \alpha}} {{ \vert {\nabla w} \vert }^{p(x,t)}} \,\mathrm{d}x\,\mathrm{d}t} } \ge0. \end{aligned} $$

Noting that $\frac{{p(x,t)}}{{p(x,t) - 1}} \ge\frac{{{p^{+} }}}{{{p^{+} } - 1}} \ge\frac{\sigma}{2} = \alpha > 1$ and applying Young’s inequality, we can estimate the integrand of ${J_{3}}$ in the following way:

12 $$ \begin{aligned}[b] & \bigl\vert {\bigl({u^{\sigma}} - {v^{\sigma}}\bigr){w^{ - \alpha}}} { \vert {\nabla w} \vert ^{p(x,t) - 2}} {\nabla u\nabla w} \bigr\vert \\ &\quad= \biggl\vert {\sigma w \int_{0}^{1} {{{\bigl(\theta u + (1 - \theta )v \bigr)}^{\sigma - 1}}\,d\theta{w^{ - \alpha}} {{ \vert {\nabla w} \vert }^{p(x,t) - 2}}\nabla u\nabla w} } \biggr\vert \\ &\quad\le\frac{C}{{{w^{\alpha}}}}\biggl[\frac{{{v^{\sigma}} + {d_{0}}}}{C}\biggr]{ \vert {\nabla w} \vert ^{p(x,t)}} + {C_{1}}\bigl(\sigma,{d_{0}},K(T),{p^{\pm}} \bigr){ \vert w \vert ^{p'(x,t)}} { \vert {\nabla u} \vert ^{p(x,t)}}] \\ &\quad= \frac{{({v^{\sigma}} + {d_{0}})}}{{{2^{{p^{+} } + 1}}{w^{\alpha}}}}{ \vert {\nabla w} \vert ^{p(x,t)}} + {C_{1}}\bigl(\sigma,{d_{0}},K(T),{p^{\pm}}\bigr){ \vert w \vert ^{p'(x,t) - \alpha }} { \vert {\nabla u} \vert ^{p(x,t)}} \\ &\quad\le\frac{{({v^{\sigma}} + {d_{0}})}}{{{2^{{p^{+} } + 1}}{w^{\alpha}}}}{ \vert {\nabla w} \vert ^{p(x,t)}} + {C_{1}}\bigl(\sigma,{d_{0}},K(T),{p^{\pm}}\bigr){ \vert {\nabla u} \vert ^{p(x,t)}}. \end{aligned} $$

Substituting (12) into $J_{3}$, we get

13 $$ {J_{3}} \le\frac{1}{2}{J_{2}} + C{ \int{ \int_{{Q_{\varepsilon,\tau}}} { \vert {\nabla u} \vert } } ^{p(x,t)}} \,\mathrm{d}x\,\mathrm {d}t. $$

Second, we consider the case $1 < {p^{-} } \le p(x,t) < 2$, ${p^{+} } \ge2$. According to the second inequality of Lemma 3.1, it is easily seen that the following inequalities hold:

14 $$ \begin{aligned}[b] {J_{2}} = \int{ \int_{{Q_{\varepsilon,\tau}}} {\bigl({v^{\sigma}} + {d_{0}} \bigr){w^{ - \alpha}}\bigl({{ \vert {\nabla w} \vert }^{p(x,t) - 2}} \nabla u - {{ \vert {\nabla v} \vert }^{p(x,t) - 2}}\nabla v\bigr)\nabla w \,\mathrm{d}x\,\mathrm{d}t} } \\ \ge\bigl({p^{-} } - 1\bigr) \int{ \int_{{Q_{\varepsilon,\tau}}} {\bigl({v^{\sigma}} + {d_{0}} \bigr){w^{ - \alpha}} {{\bigl( \vert {\nabla w} \vert + \vert {\nabla v} \vert \bigr)}^{p(x,t) - 2}} {{ \vert {\nabla w} \vert }^{2}} \,\mathrm{d}x\,\mathrm{d}t} }. \end{aligned} $$

Using the conditions $1 < \alpha \le\frac{{{p^{+} }}}{{{p^{+} } - 1}} \le2$ and Young’s inequality, we can evaluate the integrand of $J_{3}$ as follows:

15 $$ \begin{aligned}[b] & \bigl\vert {\bigl({u^{\sigma}} - {v^{\sigma}}\bigr){w^{ - \alpha}} {{ \vert {\nabla w} \vert }^{p(x,t) - 2}}\nabla u\nabla w} \bigr\vert \\ &\quad = \biggl\vert {\sigma w \int_{0}^{1} {{{\bigl(\theta u + (1 - \theta )v \bigr)}^{\sigma - 1}}\,d\theta{w^{ - \alpha}} {{ \vert {\nabla w} \vert }^{p(x,t) - 2}}\nabla u\nabla w} } \biggr\vert \\ &\quad \le\frac{{({v^{\sigma}} + {d_{0}})({p^{-} } - 1)}}{{2{w^{\alpha}}}}{\bigl( \vert {\nabla w} \vert + \vert {\nabla v} \vert \bigr)^{p(x,t) - 2}} { \vert {\nabla w} \vert ^{2}} \\ &\qquad{} + {C_{1}}\bigl(\sigma,{d_{0}},K(T),{p^{\pm}} \bigr){ \vert w \vert ^{2 - \alpha}} \vert {\nabla w} \vert + \vert { \nabla v} \vert {)^{p(x,t)}}. \end{aligned} $$

Plugging (15) into $J_{3}$, we get

16 $$ {J_{3}} \le\frac{1}{2}{J_{2}} + C \int{ \int_{{Q_{\varepsilon,\tau}}} { \vert {\nabla w} \vert + \vert {\nabla v} \vert {)^{p(x,t)}}} } \,\mathrm{d}x\,\mathrm{d}t. $$

Plugging estimates (10), (11), (13) and (10), (14), (16) into (9) and dropping the nonnegative terms, we arrive at the inequality

17 $$ (\delta - 2\varepsilon) \bigl(1 - {2^{1 - \alpha}}\bigr){\varepsilon^{1 - \alpha}} \mu({\Omega_{\delta}}) \le\tilde{C} $$

with a constant *C̃* independent of *ε*.

Notice that ${\lim_{\varepsilon \to0}}(\delta - 2\varepsilon)(1 - {2^{1 - \alpha}}){\varepsilon^{1 - \alpha}}\mu({\Omega_{\delta}}) = + \infty$, a contradiction. This means that $\mu({\Omega_{\delta}}) = 0$ and $w \le0$ a.e. in ${Q_{\tau}}$. □

### Lemma 3.3

*Let* ${u_{\varepsilon}}$ *be weak solutions of* (5). *Then*

18 $$\begin{aligned}& {u_{0\varepsilon}} \le{u_{\varepsilon}} \le{ \vert {{u_{0}}} \vert _{\infty}} + \varepsilon, \end{aligned}$$

19 $$\begin{aligned}& {u_{{\varepsilon_{1}}}} \le{u_{{\varepsilon_{2}}}} \quad \textit{for } { \varepsilon_{1}} \le{\varepsilon_{2}}. \end{aligned}$$

### Proof

First, we prove ${u_{\varepsilon}} \ge{u_{0\varepsilon}}$ by contradiction. Assume that ${u_{\varepsilon}} \le{u_{0\varepsilon}}$ in $Q_{T}^{0}$, $Q_{T}^{0} \subset {Q_{T}}$. Noting that ${u_{\varepsilon}} \ge{u_{0\varepsilon}}$ on $\partial{Q_{T}}$, we may assume that ${u_{\varepsilon}} = {u_{0\varepsilon}}$ on $\partial Q_{T}^{0}$. With (5) and letting $t = 0$, we deduce that

20 $$\begin{aligned}& L{u_{0\varepsilon}} = {\beta_{\varepsilon}}({u_{0\varepsilon}} - {u_{0\varepsilon}}) = - 1, \end{aligned}$$

21 $$\begin{aligned}& L{u_{\varepsilon}} = {\beta_{\varepsilon}}({u_{\varepsilon}} - {u_{0\varepsilon}}) \le - 1. \end{aligned}$$

From Lemma 3.2 we conclude that

22 $$ u(x,t) \le{u_{0\varepsilon}}(x) \quad \text{for any } (x,t) \in { \Omega_{T}}, $$

obtaining a contradiction.

Second, we pay attention to ${u_{\varepsilon}}(t,x) \le{| {{u_{0}}} |_{\infty}} + \varepsilon$. Applying the definition of ${\beta_{\varepsilon}}( \cdot )$, we have

23 $$ L\bigl( {{{ \vert {{u_{0}}} \vert }_{\infty}} + \varepsilon} \bigr) = 0, \quad L{u_{\varepsilon}} \le0. $$

From (5) it is easy to prove that ${u_{\varepsilon}}(x,t) \ge \varepsilon$ on $\partial\Omega \times(0,T)$ and ${u_{0\varepsilon}}(x) \ge\varepsilon$ in Ω. Thus, combining (21) and (23) and repeating Lemma 3.3, we have

24 $$ {u_{\varepsilon}}(x,t) \ge\varepsilon \quad\text{in } \Omega \times (0,T). $$

Third, we aim to prove (19). Since

25 $$\begin{aligned}& L{u_{{\varepsilon_{1}}}} - {\beta_{{\varepsilon_{1}}}}({u_{0{\varepsilon _{1}}}} - {u_{{\varepsilon_{1}}}}) = 0, \end{aligned}$$

26 $$\begin{aligned}& L{u_{{\varepsilon_{2}}}} - {\beta_{{\varepsilon_{2}}}}({u_{0{\varepsilon _{2}}}} - {u_{{\varepsilon_{2}}}}) = 0. \end{aligned}$$

It follows by ${\varepsilon_{1}} \le{\varepsilon_{2}}$ and the definition of ${\beta_{\varepsilon}}( \cdot )$ that

27 $$ \begin{aligned}[b] &{\partial_{t}} {u_{{\varepsilon_{2}}}} - L{u_{{\varepsilon_{2}}}} - {\beta _{{\varepsilon_{1}}}}({u_{0{\varepsilon_{1}}}} - {u_{{\varepsilon_{2}}}}) \\ &\quad={\beta_{{\varepsilon_{2}}}}({u_{0{\varepsilon_{2}}}} - {u_{{\varepsilon_{2}}}}) - { \beta_{{\varepsilon_{1}}}}({u_{0{\varepsilon _{1}}}} - {u_{{\varepsilon_{2}}}}) \ge{ \beta_{{\varepsilon _{2}}}}({u_{0{\varepsilon_{2}}}} - {u_{{\varepsilon_{2}}}}) - {\beta _{{\varepsilon_{1}}}}({u_{0{\varepsilon_{2}}}} - {u_{{\varepsilon_{2}}}}) \ge0. \end{aligned} $$

Thus, Lemma 3.3 can be proved by combining initial and boundary conditions in (5). □

Moreover, with (18), we assert that there exists a subsequence *ε* (still denoted by *ε*) such that

28 $$\begin{aligned}& {u_{\varepsilon}} \to u \in{L^{p}}\bigl(0,T;W_{0}^{1,p}({ \Omega_{T}})\bigr) \quad \text{as } \varepsilon \to0, \end{aligned}$$

29 $$\begin{aligned}& {u_{\varepsilon}} \ge u \ge0 \quad \text{for any } \varepsilon > 0. \end{aligned}$$

### Lemma 3.4

*Let* ${u_{\varepsilon}}$ *be a solution of problem* (5). *For any* $\varepsilon > 0$, *we have*

30 $$ { \Vert {{u_{\varepsilon}}} \Vert _{\infty,{Q_{T}}}} \le{ \Vert {{u_{0}}} \Vert _{\infty,\Omega}} + \int_{0}^{T} {{{ \bigl\Vert {f(x,t)} \bigr\Vert }_{\infty,\Omega}}\,\mathrm{d}t} = K(T) < \infty. $$

### Proof

Define

$${u_{\varepsilon M}} = \textstyle\begin{cases} M & \text{if } {u_{\varepsilon}} > M,\\ {u_{\varepsilon}} & \text{if } \vert {{u_{\varepsilon}}} \vert \le M,\\ - M &\text{if } {u_{\varepsilon}} < - M. \end{cases} $$

Choosing $u_{\varepsilon M}^{2k - 1}$ as a test-function in (8) and letting ${t_{1}} = t$ and ${t_{2}} = t + h$, we conclude that

31 $$ \begin{aligned}[b] &\frac{1}{{2k}} \int_{t}^{t + h} {\frac{\mathrm{d}}{{\mathrm{d}t}}\biggl( { \int _{\Omega}{u_{\varepsilon M}^{2k}\,dx} } \biggr)} \,\mathrm{d}t + \int_{t}^{t + h} { \int_{\Omega}{(2k - 1){a_{\varepsilon,M}}({u_{\varepsilon M}})} } u_{\varepsilon M}^{2(k - 1)}{ \vert {\nabla{u_{\varepsilon M}}} \vert ^{p(x,t)}}\,\mathrm{d}x\,\mathrm{d}t \\ &\quad = \int_{t}^{t + h} { \int_{\Omega}{fu_{\varepsilon M}^{2k - 1}\,\mathrm{d}x \,\mathrm{d}t} - \int_{t}^{t + h} { \int_{\Omega}{{\beta _{\varepsilon}}u_{\varepsilon M}^{2k - 1} \,\mathrm{d}x} } }. \end{aligned} $$

Letting $h \to0$ and applying Lebesgue’s dominated convergence theorem, we have that, for all $t \in(0,T)$,

32 $$ \begin{aligned}[b] &\frac{1}{{2k}}\frac{\mathrm{d}}{{\mathrm{d}t}} \int_{\Omega}{u_{\varepsilon M}^{2k}\,\mathrm{d}x} + \int_{\Omega}{(2k - 1){a_{\varepsilon,M}}({u_{\varepsilon M}})} u_{\varepsilon M}^{2(k - 1)}{ \vert {\nabla{u_{\varepsilon M}}} \vert ^{p(x,t)}}\,\mathrm{d}x \\ &\quad = \int_{\Omega}{fu_{\varepsilon M}^{2k - 1}\,\mathrm{d}x} - \int _{\Omega}{{\beta_{\varepsilon}}u_{\varepsilon M}^{2k - 1} \,\mathrm{d}x}. \end{aligned} $$

Using Holder’s inequality, we obtain

$$\begin{aligned}& \biggl\vert { \int_{\Omega}{fu_{\varepsilon M}^{2k - 1}\,dx} } \biggr\vert \le \bigl\Vert {{u_{\varepsilon M}}(\cdot,t)} \bigr\Vert _{2k,\Omega}^{2k - 1} \cdot{ \bigl\Vert {f(\cdot,t)} \bigr\Vert _{2k,\Omega}},\quad k = 1,2,\dots, \\& \biggl\vert { \int_{\Omega}{{\beta_{\varepsilon}}u_{\varepsilon M}^{2k - 1} \,\mathrm{d}x} } \biggr\vert \le \int_{\Omega}{u_{\varepsilon M}^{2k - 1}\,\mathrm{d}x} \le \bigl\Vert {{u_{\varepsilon M}}(\cdot,t)} \bigr\Vert _{2k,\Omega}^{2k - 1}, \end{aligned}$$

whence

33 $$ \begin{aligned}[b] & \Vert {{u_{\varepsilon M}}} \Vert _{2k,\Omega}^{2k - 1}\frac{\mathrm{d}}{{\mathrm{d}t}}\bigl({ \Vert {{u_{\varepsilon M}}} \Vert _{2k,\Omega}}\bigr) + (2k - 1) \int_{\Omega}{{a_{\varepsilon,M}}({u_{\varepsilon M}})} u_{\varepsilon M}^{2(k - 1)}{ \vert {\nabla{u_{\varepsilon,M}}} \vert ^{p(x,t)}}\,\mathrm{d}x \\ &\quad \le \Vert {{u_{\varepsilon M}}} \Vert _{2k,\Omega }^{2k - 1} \cdot{ \bigl\Vert {f( \cdot,t)} \bigr\Vert _{2k,\Omega}} + C(T) \Vert {{u_{\varepsilon M}}} \Vert _{2k,\Omega}^{2k - 1},\quad k = 1,2,\dots. \end{aligned} $$

By integration over $(0, t)$, for all *t*, we have

$${ \bigl\Vert {{u_{\varepsilon M}}(\cdot,t)} \bigr\Vert _{2k,\Omega}} \le { \bigl\Vert {{u_{\varepsilon M}}(\cdot,0)} \bigr\Vert _{2k,\Omega}} + \int_{0}^{T} {{{ \Vert f \Vert }_{2k,\Omega}} \,\mathrm{d}t} + C(T),\quad \forall k \in\mathbb{N}. $$

Then, as $k \to\infty$,

$$\begin{aligned}[b] { \bigl\Vert {{u_{\varepsilon M}}(\cdot,t)} \bigr\Vert _{\infty,\Omega }} &\le{ \bigl\Vert {{u_{\varepsilon M}}(\cdot,0)} \bigr\Vert _{\infty,\Omega}} + \int_{0}^{T} {{{ \Vert f \Vert }_{\infty,\Omega}} \,\mathrm{d}t} \\ &\le{ \Vert {{u_{0}}} \Vert _{\infty,\Omega}} + \int_{0}^{T} {{{ \Vert f \Vert }_{\infty,\Omega}} \,\mathrm{d}t} + C(T) = K(T). \end{aligned} $$

 □

If we chose $M > K(T)$ then ${u_{\varepsilon M}}(\cdot,t) \le\sup| {{u_{\varepsilon M}}(\cdot,t)} | \le K(T) < M$, and therefore ${u_{\varepsilon M}}(\cdot,t) = {u_{\varepsilon}}(\cdot,t)$.

### Corollary 3.1

*Choosing* *M* *large enough*, *we have*

$$\min\bigl\{ u_{\varepsilon}^{2},{M^{2}}\bigr\} = u_{\varepsilon}^{2} \quad \textit{and}\quad {a_{\varepsilon,M}} \bigl(u_{\varepsilon M}\bigr) = {a_{\varepsilon,M}}({a_{\varepsilon}}) = \bigl({\varepsilon^{2}} + u_{\varepsilon}^{2} \bigr)^{\sigma/ 2} + {d_{0}}. $$

### Corollary 3.2

*If* ${u_{0}} \ge0$ *and* $f \ge0$, *then the solution* ${u_{\varepsilon}}(x,t)$ *is nonnegative in* ${Q_{T}}$.

### Proof

Set $u_{\varepsilon}^{-} = \min\{ {u_{\varepsilon}},0\} $. Then $u_{\varepsilon}^{-} (x,0) = 0$, $u_{\varepsilon}^{-} | {{\Gamma_{T}}} = 0$, and

$$\frac{1}{2}\frac{\mathrm{d}}{{\mathrm{d}t}}\bigl( \bigl\Vert {u_{\varepsilon}^{-} (x,t)} \bigr\Vert _{2,\Omega}^{2}\bigr) + \int_{\Omega}{{a_{\varepsilon,M}}({u_{\varepsilon}})} { \bigl\vert {\nabla u_{\varepsilon}^{-} } \bigr\vert ^{p(x,t)}}\,\mathrm{d}x \le0. $$

It follows that, for every $t > 0$,

$$\bigl\Vert {u_{\varepsilon}^{-} (x,t)} \bigr\Vert _{2,\Omega} \le \bigl\Vert {u_{\varepsilon}^{-} (\cdot,0)} \bigr\Vert _{2,\Omega} = 0, $$

and the required assertion follows. □

### Lemma 3.5

*The solution of problem* (5) *satisfies the estimates*

34 $$\begin{aligned}& { \int{ \int_{{Q_{T}}} {u_{\varepsilon}^{\sigma}{{ \vert {\nabla {u_{\varepsilon}}} \vert }^{p(x,t)}}\,\mathrm{d}x\,\mathrm{d}t \le K(T) \vert \Omega \vert } } ^{\frac{1}{2}}}, \end{aligned}$$

35 $$\begin{aligned}& {\varepsilon^{\sigma}} { \int{ \int_{{Q_{T}}} {{{ \vert {\nabla {u_{\varepsilon}}} \vert }^{p(x,t)}}\,\mathrm{d}x\,\mathrm{d}t \le K(T) \vert \Omega \vert } } ^{\frac{1}{2}}}, \end{aligned}$$

36 $$\begin{aligned}& {d_{0}} { \int{ \int_{{Q_{T}}} {{{ \vert {\nabla{u_{\varepsilon}}} \vert }^{p(x,t)}}\,\mathrm{d}x\,\mathrm{d}t \le K(T) \vert \Omega \vert } } ^{\frac{1}{2}}}. \end{aligned}$$

### Proof

Similarly as in Lemma 3.4, we take $k = 1$ in (32) to get

$$\frac{\mathrm{d}}{{\mathrm{d}t}}{ \bigl\Vert {{u_{\varepsilon}}(\cdot,t)} \bigr\Vert _{2,\Omega}} + \int_{\Omega}{{a_{\varepsilon,M}}({u_{\varepsilon}}){{ \vert {\nabla{u_{\varepsilon}}} \vert }^{p(x,t)}}\,\mathrm{d}x} \le{ \Vert f \Vert _{2,\Omega}} + C(T),\quad \forall t \in(0,T). $$

Clearly, integrating over $(0,t)$, we have

37 $$ { \bigl\Vert {{u_{\varepsilon}}(\cdot,t)} \bigr\Vert _{2,\Omega}} + \int _{0}^{t} { \int_{\Omega}{{a_{\varepsilon,M}}({u_{\varepsilon}}){{ \vert { \nabla{u_{\varepsilon}}} \vert }^{p(x,t)}}\,\mathrm{d}x} \,\mathrm{d}t \le} { \bigl\Vert {{u_{\varepsilon}}(\cdot,t)} \bigr\Vert _{2,\Omega}} + \int_{0}^{T} {{{ \Vert f \Vert }_{2,\Omega }} \,\mathrm{d}t} + C(T). $$

Note that the first term on the left-hand side is nonnegative. We drop the nonpositive term in (37) to get

$$\int_{0}^{t} { \int_{\Omega}{{a_{\varepsilon,M}}({u_{\varepsilon}}){{ \vert { \nabla{u_{\varepsilon}}} \vert }^{p(x,t)}}\,\mathrm{d}x} \,\mathrm{d}t \le} K(T){ \vert \Omega \vert ^{\frac{1}{2}}}. $$

If ${a_{\varepsilon,M}}({u_{\varepsilon}}) \ge{d_{0}}$, then we have inequality (36), and if ${a_{\varepsilon,M}}({u_{\varepsilon}}) \ge{\varepsilon^{\sigma}}$, then we have inequality (35) such that $M > K(T)$, and we have ${a_{\varepsilon,M}}({u_{\varepsilon}}) \ge u_{\varepsilon}^{\sigma}$. Furthermore, we get inequality (34). □

### Lemma 3.6

*The solution of problem* (5) *satisfies the estimate*

38 $$ { \Vert {{u_{\varepsilon t}}} \Vert _{W'({Q_{T}})}} \le C\bigl( { \sigma,{p^{\pm}},K(T), \vert \Omega \vert } \bigr). $$

### Proof

From identity (7) we get

$$\begin{aligned}[b] \int{ \int_{{Q_{T}}} {{u_{\varepsilon t}}\xi\,\mathrm{d}x\,\mathrm{d}t} } &= - \int{ \int_{{Q_{T}}} {\bigl[{{\bigl(u_{\varepsilon}^{\sigma}+ { \varepsilon ^{2}}\bigr)}^{{\sigma / 2}}} + {d_{0}}\bigr]{{ \vert {\nabla{u_{\varepsilon}}} \vert }^{p(x,t) - 2}}} } \nabla{u_{\varepsilon}}\nabla\xi\,\mathrm {d}x\,\mathrm{d}t \\ &\quad{}+ \int{ \int_{{Q_{T}}} {f(x,t)\xi(x,t)\,\mathrm{d}x\,\mathrm{d}t} } - \int{ \int_{{Q_{T}}} {{\beta_{\varepsilon}}(x,t)\xi(x,t)\,\mathrm {d}x \,\mathrm{d}t} }. \end{aligned} $$

Applying the fact that ${\beta_{\varepsilon}}(x,t) \in(0,1)$, we get

$$\begin{aligned} \int{ \int_{{Q_{T}}} {{u_{\varepsilon t}}\xi\,\mathrm{d}x\,\mathrm{d}t} } &\le \int_{0}^{T} { \int_{\Omega}{\bigl[{{\bigl(u_{\varepsilon}^{\sigma}+ { \varepsilon ^{2}}\bigr)}^{{\sigma / 2}}} + {d_{0}}\bigr]{{ \vert {\nabla{u_{\varepsilon}}} \vert }^{p(x,t) - 1}}} } \nabla{u_{\varepsilon}}\nabla\xi\,\mathrm {d}x\,\mathrm{d}t\\ &\quad {} + \int_{0}^{T} { \int_{\Omega}{ \vert {f + 1} \vert \cdot \vert \xi \vert } } \,\mathrm{d}x\,\mathrm{d}t. \end{aligned} $$

Using the Hölder inequality repeatedly, we have that

$$\begin{aligned}[b] \int{ \int_{{Q_{T}}} {{u_{\varepsilon t}}\xi\,\mathrm{d}x\,\mathrm{d}t} } & \le2{ \bigl\Vert {\bigl[{{\bigl(u_{\varepsilon}^{\sigma}+ {\varepsilon ^{2}}\bigr)}^{{\sigma / 2}}} + {d_{0}}\bigr]{{ \vert { \nabla{u_{\varepsilon}}} \vert }^{p(x,t) - 1}}} \bigr\Vert _{p'(x,t)}} { \Vert {\nabla\xi} \Vert _{p(x,t)}}\\ &\quad {} + 2{ \Vert {f + 1} \Vert _{p'(x,t)}} \cdot{ \Vert \xi \Vert _{p(x,t)}} \\ &\le2\max\{ {F_{1}},{F_{2}}\} { \Vert {\nabla\xi} \Vert _{p(x,t)}} + 2\max\{ {{F_{3}},{F_{4}}} \}{ \Vert \xi \Vert _{p(x,t)}} \\ & \le\bigl(2{\bigl({\bigl({K^{2}}(T) + 1\bigr)^{{\sigma / 2}}} + {d_{0}}\bigr)^{\frac{1}{{{p^{\pm}} - 1}}}}K(T) \vert \Omega \vert + 2{ \vert {f + 1} \vert _{\infty}} \vert T \vert \bigr){ \Vert \xi \Vert _{W({Q_{T}})}}, \end{aligned} $$

where

$$\begin{aligned}& {F_{1}} = {\biggl( { \int_{0}^{T} { \int_{\Omega}{{{\bigl\{ {\bigl[{{\bigl(u_{\varepsilon}^{\sigma}+ {\varepsilon^{2}}\bigr)}^{{\sigma / 2}}} + {d_{0}}\bigr]{{ \vert {\nabla {u_{\varepsilon}}} \vert }^{p(x,t) - 1}}} \bigr\} }^{\frac {{p(x,t)}}{{p(x,t) - 1}}}}\,\mathrm{d}x\,\mathrm{d}t} } } \biggr)^{\frac{1}{{p' + }}}}, \\& {F_{2}} = {\biggl( { \int_{0}^{T} { \int_{\Omega}{{{\bigl\{ {\bigl[{{\bigl(u_{\varepsilon}^{\sigma}+ {\varepsilon^{2}}\bigr)}^{{\sigma / 2}}} + {d_{0}}\bigr]{{ \vert {\nabla {u_{\varepsilon}}} \vert }^{p(x,t) - 1}}} \bigr\} }^{\frac {{p(x,t)}}{{p(x,t) - 1}}}}\,\mathrm{d}x\,\mathrm{d}t} } } \biggr)^{\frac{1}{{p' - }}}}, \\& {F_{3}} = {\biggl( { \int_{0}^{T} { \int_{\Omega}{{{ \vert f \vert }^{p'(x,t)}}\,\mathrm{d}x \,\mathrm{d}t} } } \biggr)^{\frac{1}{{p' + }}}}, \qquad {F_{4}} = {\biggl( { \int_{0}^{T} { \int_{\Omega}{{{ \vert {f + 1} \vert }^{p'(x,t)}} \,\mathrm{d}x\,\mathrm{d}t} } } \biggr)^{\frac{1}{{p' - }}}}. \end{aligned}$$

Then (38) follows from Lemma 3.5. □

From [6] we can get the following inclusions:

$$\begin{aligned}& {u_{\varepsilon}} \in W({Q_{T}}) \subseteq{L^{{p^{-} }}} \bigl(0,T;W_{0}^{1,{p^{-} }}(\Omega)\bigr), \qquad {u_{\varepsilon t}} \in W'({Q_{T}}) \subseteq {L^{\frac{{{p^{+} }}}{{{p^{+} } - 1}}}}\bigl(0,T;{V_{+} }(\Omega)\bigr), \\& W_{0}^{1,{p^{-} }}(\Omega) \subset{L^{2}}(\Omega) \subset{V_{+} }^{\prime}(\Omega) \quad \text{with } {V_{+} }(\Omega) = \bigl\{ u(x)\vert {u \in{L^{2}}(\Omega) \cap W_{0}^{1,1}( \Omega)}, \vert {\nabla u} \vert \in{L^{{p^{+} }}}\bigr\} . \end{aligned}$$

These conclusions, together with the uniform estimates in *ε*, allow us to extract from the sequence $\{ {u_{\varepsilon}}\} $ a subsequence (for simplicity, we assume that it merely coincides with the whole sequence) such that

39 $$ \textstyle\begin{cases} {u_{\varepsilon}} \to u & \mbox{a.e. in }{Q_{T}},\\ \nabla{u_{\varepsilon}} \to\nabla u &\text{weakly in }{L^{p(x,t)}}({Q_{T}}),\\ u_{\varepsilon}^{\sigma}{ \vert {\nabla{u_{\varepsilon}}} \vert ^{p(x,t) - 2}}{D_{i}}{u_{\varepsilon}} \to{A_{i}}(x,t) & \text{weakly in } {L^{p'(x,t)}}({Q_{T}}),\\ { \vert {\nabla{u_{\varepsilon}}} \vert ^{p(x,t) - 2}}{D_{i}}{u_{\varepsilon}} \to{W_{i}}(x,t) & \text{weakly in }{L^{p'(x,t)}}({Q_{T}}) \end{cases} $$

for some functions $u \in W({Q_{T}})$, ${A_{i}}(x,t) \in {L^{p'(x,t)}}({Q_{T}})$, and ${W_{i}}(x,t) \in{L^{p'(x,t)}}({Q_{T}})$.

### Lemma 3.7

*For almost all* $(x,t) \in{Q_{T}}$,

$$\lim_{\varepsilon \to{0^{+} }} \int{ \int_{{Q_{T}}} {\bigl({{\bigl(u_{\varepsilon}^{2} + { \varepsilon^{2}}\bigr)}^{\frac{\sigma}{2}}} - u_{\varepsilon}^{\sigma}\bigr){{ \vert {\nabla{u_{\varepsilon}}} \vert }^{p(x,t) - 2}} \nabla{u_{\varepsilon}}\nabla\xi\,\mathrm{d}x\,\mathrm{d}t = 0,\quad \forall\xi = W({Q_{T}})} }. $$

### Proof

We first compute

$$\begin{aligned}[b] I &\stackrel{\Delta}{=} \int{ \int_{{Q_{T}}} {\bigl({{\bigl(u_{\varepsilon}^{2} + { \varepsilon^{2}}\bigr)}^{\frac{\sigma}{2}}} - u_{\varepsilon}^{\sigma}\bigr){{ \vert {\nabla{u_{\varepsilon}}} \vert }^{p(x,t) - 2}}\nabla {u_{\varepsilon}}\nabla\xi\,\mathrm{d}x\,\mathrm{d}t} } \\ & = \frac{\sigma}{2}{\varepsilon^{2}} \int{ \int_{{Q_{T}}} {\biggl( { \int_{0}^{1} {{{\bigl(u_{\varepsilon}^{2} + s{\varepsilon^{2}}\bigr)}^{\frac{{\sigma - 2}}{2}}}\,ds} } \biggr)} } { \vert { \nabla{u_{\varepsilon}}} \vert ^{p(x,t) - 2}}\nabla{u_{\varepsilon}}\nabla \xi\,\mathrm{d}x\,\mathrm{d}t \\ & \le\sigma{\varepsilon^{2}} {\bigl({K^{2}}(T) + 1 \bigr)^{{\frac{{\sigma - 2}}{2}}}} { \bigl\Vert {{{ \vert {\nabla{u_{\varepsilon}}} \vert }^{p(x,t) - 1}}} \bigr\Vert _{p'(x,t)}} { \Vert {\nabla\xi } \Vert _{p(x,t)}} \\ & \le C{\varepsilon^{2}}\max\biggl\{ {{{\biggl( \int{ \int_{{Q_{T}}} {{{ \vert {\nabla{u_{\varepsilon}}} \vert }^{p(x,t)}}\,\mathrm{d}x\,\mathrm{d}t} } \biggr)}^{\frac{{{p^{+} } - 1}}{{{p^{+} }}}}},{{\biggl( \int{ \int_{{Q_{T}}} {{{ \vert {\nabla {u_{\varepsilon}}} \vert }^{p(x,t)}}\,\mathrm{d}x\,\mathrm{d}t} } \biggr)}^{\frac{{{p^{-} } - 1}}{{{p^{-} }}}}}} \biggr\} { \Vert {\nabla\xi} \Vert _{p(x,t)}}. \end{aligned} $$

By (35) we get

$$I \le C{\varepsilon^{2 - \frac{{\sigma({p^{+} } - 1)}}{{{p^{+} }}}}} { \Vert {\nabla\xi} \Vert _{p(x,t)}}. $$

Passing to the limit as $\varepsilon \to0$, we obtain Lemma 3.7. □

### Lemma 3.8

*For almost all* $(x,t) \in{Q_{T}}$, *we have*

$${A_{i}}(x,t) = {u^{\sigma}} {W_{i}}(x,t),\quad i = 1,2, \dots,N. $$

### Proof

In (39), letting $\varepsilon \to0$, we have

40 $$\begin{aligned}& { \int \int_{{Q_{T}}} {u_{\varepsilon}^{\sigma} \vert {\nabla {u_{\varepsilon}}} \vert } ^{p(x,t) - 2}}\nabla{u_{\varepsilon}}\nabla \xi\,\mathrm{d}x\,\mathrm{d}t \to\sum{ \int{ \int_{{Q_{T}}} {{A_{i}}(x,t){D_{i}}\xi \,\mathrm{d}x\,\mathrm{d}t} } }, \end{aligned}$$

41 $$\begin{aligned}& { \int \int_{{Q_{T}}} {u_{\varepsilon}^{\sigma} \vert {\nabla {u_{\varepsilon}}} \vert } ^{p(x,t) - 2}}\nabla{u_{\varepsilon}}\nabla \xi\,\mathrm{d}x\,\mathrm{d}t \to\sum{ \int{ \int_{{Q_{T}}} {{W_{i}}(x,t){D_{i}}\xi \,\mathrm{d}x\,\mathrm{d}t} } }. \end{aligned}$$

By Lebesgue’s dominated convergence theorem we have

42 $$ \lim_{\varepsilon \to0} \sum_{i = 1}^{N} { \int{ \int_{{Q_{T}}} {\bigl(u_{\varepsilon}^{\sigma}- {u^{\sigma}}\bigr){A_{i}}(x,t){D_{i}}\xi\,\mathrm {d}x \,\mathrm{d}t = 0} }.} $$

So

$$\begin{aligned}[b] &\lim_{\varepsilon \to0} \sum{ \int{ \int_{{Q_{T}}} {\bigl(u_{\varepsilon}^{\sigma}{{ \vert { \nabla{u_{\varepsilon}}} \vert }^{p(x,t) - 2}} {D_{i}} {u_{\varepsilon}} - {u^{\sigma}} {W_{i}}(x,t) \bigr){D_{i}}\xi\,\mathrm {d}x\,\mathrm{d}t} } } \\ &\quad= \lim_{\varepsilon \to0} \int{ \int_{{Q_{T}}} {\bigl(u_{\varepsilon}^{\sigma}- {u^{\sigma}}\bigr){{ \vert {\nabla{u_{\varepsilon}}} \vert }^{p(x,t) - 2}} {D_{i}} {u_{\varepsilon}} + {u^{\sigma}} \bigl({{ \vert {\nabla{u_{\varepsilon}}} \vert }^{p(x,t) - 2}} {D_{i}} {u_{\varepsilon}} - {W_{i}}(x,t) \bigr){D_{i}}\xi\,\mathrm{d}x\,\mathrm{d}t} }\\ &\quad = 0. \end{aligned} $$

By (40)–(42) and the previous inequalities, we complete the proof of Lemma 3.8. □

### Lemma 3.9

*For almost all* $(x,t) \in{Q_{T}}$, *we have*

$${W_{i}}(x,t) = { \vert {\nabla{u_{\varepsilon}}} \vert ^{p(x,t) - 2}} {D_{i}}u,\quad i = 1,2,\dots,N. $$

### Proof

In (8), choosing $\xi = ({u_{\varepsilon}} - u)\Phi$ with $\Phi \in W({Q_{T}})$, $\Phi \ge0$, we have

$$\begin{aligned}[b] & \int{ \int_{{Q_{T}}} {\bigl[{u_{\varepsilon t}}({u_{\varepsilon}} - u) \Phi + \Phi\bigl(u_{\varepsilon}^{\sigma}+ {d_{0}}\bigr){{ \vert {\nabla {u_{\varepsilon}}} \vert }^{p(x,t) - 2}} \nabla{u_{\varepsilon}}\nabla({u_{\varepsilon}} - u)\bigr]\,\mathrm{d}x \,\mathrm{d}t} } \\ &\quad{} + \int{ \int_{{Q_{T}}} {\bigl[({u_{\varepsilon}} - u) \bigl(u_{\varepsilon}^{\sigma}+ {d_{0}}\bigr){{ \vert { \nabla{u_{\varepsilon}}} \vert }^{p(x,t) - 2}}\nabla{u_{\varepsilon}} \nabla\Phi - f(x,t) ({u_{\varepsilon}} - u)\Phi\bigr]\,\mathrm{d}x\,\mathrm{d}t} } \\ &\quad{}+ \int{ \int_{{Q_{T}}} {\bigl({{\bigl(u_{\varepsilon}^{\sigma}- { \varepsilon^{2}}\bigr)}^{\frac{\sigma}{2}}} - u_{\varepsilon}^{\sigma}\bigr){{ \vert {\nabla{u_{\varepsilon}}} \vert }^{p(x,t) - 2}}\nabla {u_{\varepsilon}}\nabla\xi\,\mathrm{d}x\,\mathrm{d}t} } = 0. \end{aligned} $$

It follows that

43 $$ \lim_{\varepsilon \to0} \int{ \int_{{Q_{T}}} {\Phi\bigl(u_{\varepsilon}^{\sigma}+ {d_{0}}\bigr){{ \vert {\nabla{u_{\varepsilon}}} \vert }^{p(x,t) - 2}}\nabla{u_{\varepsilon}}\nabla({u_{\varepsilon}} - u) \,\mathrm{d}x\,\mathrm{d}t = 0.} } $$

On the other hand, from ${u_{\varepsilon}},u \in{L^{\infty}}({Q_{T}})$ and $| {\nabla u} | \in {L^{p(x,t)}}({Q_{T}})$ we get

44 $$\begin{aligned}& \lim_{\varepsilon \to0} \int{ \int_{{Q_{T}}} {\Phi\bigl(u^{\sigma}+ {d_{0}}\bigr){{ \vert {\nabla u} \vert }^{p(x,t) - 2}}\nabla u \nabla({u_{\varepsilon}} - u)\,\mathrm{d}x\,\mathrm{d}t = 0} }, \end{aligned}$$

45 $$\begin{aligned}& \lim_{\varepsilon \to0} \int{ \int_{{Q_{T}}} {\Phi\bigl(u_{\varepsilon}^{\sigma}+ {u^{\sigma}}\bigr){{ \vert {\nabla{u_{\varepsilon}}} \vert }^{p(x,t) - 2}}\nabla{u_{\varepsilon}}\nabla({u_{\varepsilon}} - u) \,\mathrm{d}x\,\mathrm{d}t = 0.} } \end{aligned}$$

Note that

46 $$ \begin{aligned}[b] 0 &\le\bigl({ \vert {\nabla{u_{\varepsilon}}} \vert ^{p(x,t) - 2}}\nabla{u_{\varepsilon}} - { \vert {\nabla u} \vert ^{p(x,t) - 2}}\nabla u\bigr)\nabla({u_{\varepsilon}} - u) \\ &\le\frac{1}{{{d_{0}}}}\bigl[\bigl(u_{\varepsilon}^{\sigma}+ {d_{0}}\bigr){ \vert {\nabla{u_{\varepsilon}}} \vert ^{p(x,t) - 2}}\nabla {u_{\varepsilon}} - \bigl(u_{\varepsilon}^{\sigma}- u^{\sigma}\bigr){ \vert {\nabla{u_{\varepsilon}}} \vert ^{p(x,t) - 2}}\nabla u\bigr]\nabla ({u_{\varepsilon}} - u) \\ &\quad{} - \frac{1}{{{d_{0}}}}\bigl(u_{\varepsilon}^{\sigma}+ {d_{0}}\bigr){ \vert {\nabla{u_{\varepsilon}}} \vert ^{p(x,t) - 2}}\nabla u\nabla({u_{\varepsilon}} - u). \end{aligned} $$

By (44)–(46) we have

47 $$ \lim_{\varepsilon \to0} \int{ \int_{{Q_{T}}} {\Phi\bigl({{ \vert {\nabla{u_{\varepsilon}}} \vert }^{p(x,t) - 2}}\nabla {u_{\varepsilon}} - } } { \vert { \nabla{u_{\varepsilon}}} \vert ^{p(x,t) - 2}}\nabla u\bigr) \nabla({u_{\varepsilon}} - u)\,\mathrm {d}x\,\mathrm{d}t = 0. $$

 □

### Lemma 3.10

*As* $\varepsilon \to0$,*we have*

48 $$ {\beta_{\varepsilon}}({u_{\varepsilon}} - {u_{0}}) \to\xi \in G(u - {u_{0}}). $$

### Proof

Using (7) and the definition of ${\beta_{\varepsilon}}$, we have

$${\beta_{\varepsilon}}({u_{\varepsilon}} - {u_{0}}) \to\xi\quad \text{as } \varepsilon \to0. $$

Now we prove that $\xi \in G(u - {u_{0}})$. According to the definition of $G( \cdot)$, we only need to prove that if $u({x_{0}},{t_{0}}) > {u_{0}}({x_{0}})$, then $\xi({x_{0}},{t_{0}}) = 0$. In fact, if $u({x_{0}},{t_{0}}) > {u_{0}}(x)$, there exist a constant $\lambda > 0$ and a *δ* neighborhood ${B_{\delta}}({x_{0}},{t_{0}})$ such that if *ε* is small enough, we have

$${u_{\varepsilon}}(x,t) \ge{u_{0}}(x) + \lambda, \quad \forall(x,t) \in {B_{\delta}}({x_{0}},{t_{0}}). $$

Thus, if *ε* is small enough, then we have

$$0 \ge{\beta_{\varepsilon}}({u_{\varepsilon}} - {u_{0}}) \ge{\beta _{\varepsilon}}(\lambda) = 0, \quad \forall(x,t) \in{B_{\delta}}({x_{0}},{t_{0}}). $$

Furthermore, it follows by $\varepsilon \to0$ that

$$\xi(x,t) = 0, \quad \forall(x,t) \in{B_{\delta}}({x_{0}},{t_{0}}). $$

Hence, (48) holds, and the proof of Lemma 3.10 is completed. □

Applying (28), (29), and Lemma 3.10, it is clear that

$$u(x,t) \le{u_{0}}(x) \quad\text{in } {\Omega_{T}}, \qquad u(x,0) = {u_{0}}(x) \quad\text{in } \Omega, \xi \in G(u - {u_{0}}), $$

and thus (a), (b), and (c) hold. The remaining arguments of the existence part are the same as those of Theorem 2.1 in [8], and we omit the details. Moreover, the uniqueness of solutions can be proved by repeating Lemma 3.1.
